# Variable Admittance Control Based on Fuzzy Reinforcement Learning for Minimally Invasive Surgery Manipulator

**DOI:** 10.3390/s17040844

**Published:** 2017-04-12

**Authors:** Zhijiang Du, Wei Wang, Zhiyuan Yan, Wei Dong, Weidong Wang

**Affiliations:** State Key Laboratory of Robotics and System, Harbin Institute of Technology, 2 Yikuang Street, Harbin 150080, China; duzj01@hit.edu.cn (Z.D.); weiw230@163.com (W.W.); yanzhiyuan@163.com (Z.Y.); dongwei@hit.edu.cn (W.D.)

**Keywords:** minimally invasive surgical robot, physical human-robot interaction, reinforcement learning, variable admittance control

## Abstract

In order to get natural and intuitive physical interaction in the pose adjustment of the minimally invasive surgery manipulator, a hybrid variable admittance model based on Fuzzy Sarsa(λ)-learning is proposed in this paper. The proposed model provides continuous variable virtual damping to the admittance controller to respond to human intentions, and it effectively enhances the comfort level during the task execution by modifying the generated virtual damping dynamically. A fuzzy partition defined over the state space is used to capture the characteristics of the operator in physical human-robot interaction. For the purpose of maximizing the performance index in the long run, according to the identification of the current state input, the virtual damping compensations are determined by a trained strategy which can be learned through the experience generated from interaction with humans, and the influence caused by humans and the changing dynamics in the robot are also considered in the learning process. To evaluate the performance of the proposed model, some comparative experiments in joint space are conducted on our experimental minimally invasive surgical manipulator.

## 1. Introduction

Compared with the traditional minimally invasive treatment, robot-assisted minimally invasive surgical technology has better hand-eye coordination, field of vision, flexibility, and stability. The minimally invasive surgical robot can significantly shorten operation time and improve the quality of operations [1,2]. In preoperative preparation of the robot-assisted minimally invasive surgery, the medical staff adjusts the manipulator’s initial configuration and selects a proper port location according to the patient’s individual characteristics. The purpose of the configuration adjustment is not only to maximize the coverage rate of the workspace in the operation area, but also to minimize the intraoperative interference among manipulators [3]. Usually, the adjustment process mentioned above is done in an active compliance control according to the inferred human intention, and this physical contact between the human and the robot during their interaction is known as physical human-robot interaction (PHRI) [4].

In recent years, a number of models and algorithms for PHRI have been proposed, such as virtual-tool control [5,6], force-free control [7,8], etc. However, in practice, the most commonly used control scheme is impedance control [9]. It is a simplified model for the dynamic characteristics of the manipulator, and the model parameters can be regulated according to the specific task. Generally, it is difficult to obtain intuitive and compliant human-robot interaction with fixed parameter models during task execution. As a result, researchers have recently focused on finding an appropriate parameter modulation strategy to adjust the impedance characteristic of the robot dynamically. In [10], a discrete variable impedance control scheme was applied to the manual welding, the discrete virtual damping value was determined by the velocity threshold. Similarly, a variable admittance law based on desired velocity and acceleration was also proposed in [11] and a variable admittance model based on the end effector velocity was proposed for the redundancy manipulator [12]. In [13], a virtual damping adjustment algorithm was given by inferring human intention from the time derivative of the contact force. These methods infer human intentions through the state variables (velocity, acceleration, or force), and rely on the experience or the data collected from the physical human-robot interaction process, which limits the expansion to other motion profiles.

References [14,15,16,17,18] regulated the control model parameters based on the estimation of the human hand-arm impedance. In [14], for human-augmentation tasks, the maximum human arm stiffness during the cooperation was estimated offline. Similar to this, a real-time estimation of the human arm stiffness was taken into account for the virtual damping modification in [17], and an arm stiffness estimation method based on EMG signal was proposed in [18], the control gains were modified according to the activation level of the arm muscles. However, these estimation models do not take individual behaviors into account, and the parameter turning is sensitive to environmental interference.

For the purpose of imitating the human impedance modulation manner, some methods have been proposed on the basis of the minimum jerk trajectory model [19,20,21]. In [22,23,24], a kind of human adaptive mechatronics is presented to take the human part of the controller into account, which regulated the impedance of the robot according to the identified human dynamics model. Most of these techniques require a priori knowledge of the movement characteristics or human dynamics model and do not focus on the optimization of the overall long-term performance in the physical human-robot interaction.

Recently, learning techniques were also used to adapt the robot’s impedance or teach variable stiffness tasks to the robots through interaction with humans or environments [25,26,27]. In [28], an online impedance parameters adjustment method was proposed, which computed the optimal parameters by minimizing a cost function. In [29], a human-robot interaction system was presented to assist humans in performing a given task with minimum effort, and the optimization of the impedance parameter was transformed into a linear quadratic regulator problem which optimized the overall system performance by online learning. In our paper, a novel variable admittance controller is proposed for the manipulator configuration adjustment in joint space. Fuzzy Sarsa(λ)-learning is employed in the parameter adjustment for establishing the human part of the control model by online learning, and the virtual damping is constantly modified by minimizing a smooth performance metrics during the interaction. The parameter modulation scheme not only satisfies the requirements of the damping at various stages in the process, but also enhances the comfort level perceived by the operator during task execution. 

In this paper, a novel variable admittance controller is presented for physical human-robot interaction in joint space. Unlike the traditional teaching-playback method, the proposed joint controllers are independent of each other, which is convenient for adjusting the manipulator attitude individually. In order to improve the comfort level of the traditional variable admittance controller, a parameter modulation strategy based on Fuzzy Sarsa(λ)-learning is proposed. No prior knowledge is required for this strategy, and the individual behaviors during the interaction are taken into consider through online learning technology, which can adapt to the environment by trial and error. The inappropriate model parameters caused by personal operation habits can be modified for achieving smooth and natural behavior in the task execution.

The rest of this paper is organized as follows. The Sarsa(λ)-learning algorithms are briefly discussed in Section 2, and then the overall structure of the control scheme and the desired goal of the reinforcement learning are proposed in Section 3. Section 4 presents the experimental evaluation of the proposed control strategy on our experimental minimally invasive surgical manipulator. Finally, a discussion of the results and the conclusion are drawn in Section 5 and Section 6, respectively.

## 2. Sarsa(λ)-Algorithm

Reinforcement learning is an important branch of machine learning, and it can be used to solve problems in which actions are applied to a system over an extended period of time, in order to achieve a desired goal [30]. Therefore, it is a kind of online learning technology which can actively adapt to the environment through trial and error. In the learning process, the agent tries to find a strategy that yields high accumulated reward from interaction with an environment. Supposing that the agent is in state *s_t_* at time *t*, and an action *a_t_* is executed according to the current policy π. Then it receives a scalar reward *r*(*s_t_,a_t_*) from the environment and arrives at a new state. Next, the action values *Q*(*s_t_,a_t_*) that quantify the quality of the selected action in the current state are updated by the Bellman Optimality Equation. Repeat the above steps, and then the policy will be gradually improved based on the action values for each step of the episode until convergence.

Sarsa(λ)-learning is a well-known model-free reinforcement learning algorithm which has the merits of one-step temporal-difference learning and Monte-Carlo algorithm [31]. This is achieved by the introduction of the so-called eligibility trace, which is a temporary record of the recently visited state-action pair. An accumulating eligibility trace is given by Equation (1)

(1) $$e_{t}(s,a)=\{\begin{array}{cc} \gamma \lambda e_{t-1}(s,a)+1 & if\;s=s_{t}\;and\;a=a_{t}\\ \gamma \lambda e_{t-1}(s,a) & otherwise \end{array}$$

where *λ* is the eligibility decay rate that determines the number of the state-action pairs updated, and *γ* is a discount factor that weights the effect of the future rewards. For each state *s* ∈ *S*, the eligibility trace is incremented when the state-action pair is visited and decreases exponentially otherwise. Correspondingly, on each iteration, all the action values are updated in proportion to their eligibility traces as follows:

(2) $$Q_{t+1}(s_{t},a_{t})=Q_{t}(s_{t},a_{t})+\alpha \delta_{t}e_{t}(s_{t},a_{t}),$$

(3) $$\delta_{t}=r(s_{t},a_{t})+\gamma Q_{t}(s_{t+1},a_{t+1})-Q_{t}(s_{t},a_{t})$$

where *δ* is the temporal-difference error, and *α* is the learning rate that determines the learning speed. The policy improvement can be done is many approaches. In this paper, the Boltzmann exploration strategy is adopted for the action selection, and its general form is as follows:

(4) $$P(a_{t}|s_{t})=\frac{e^{Q_{t}(s_{t},a_{t})/\eta }}{\sum_{i=1}e^{Q_{t}(s_{t},a_{i})/\eta }},$$

where *P*(*a_t_|s_t_*) represents the probability of the action selection, and *η* is the temperature parameter, it controls the randomness of the exploration.

## 3. Variable Admittance Control Scheme

**Figure 1:**
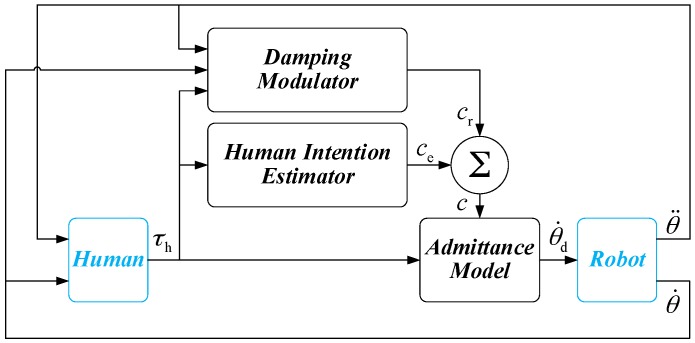
Variable admittance controller.

In this section, the overall structure of the proposed variable admittance controller is presented in detail. As shown in Figure 1, the controller consists of three parts, i.e., Admittance Model, Human Intention Estimator, and Damping Modulator. The admittance controller that allows compliant behavior of the manipulator in joint space, which establishes the dynamic relationship between interaction torque (exerted by operators) and the motion of the robot. The parameter of the admittance controller can be changed by the Human Intention Estimator and Damping Modulator during physical human-robot interaction. In order to overcome the shortcomings of the fixed admittance model, the Human Intention Estimator is used to generate the parameter of admittance controller by estimating the operator’s intention. However, the Human Intention Estimator just predicts the general trend of the human intention, sometimes, the output of the estimator is not accurate enough and the comfort level during the task execution cannot be guaranteed. For this reason, a modulator is proposed to regulate the parameter of admittance controller (output of the Human Intention Estimator) to achieve a smooth and natural behavior during the physical human-robot interaction. The individual behaviors during the interaction are taken into consideration through a modulation strategy based on Fuzzy Sarsa(λ)-learning. With continual interaction and iteration, the smoothness of the physical human-robot interaction is improved over time.

### 3.1. Variable Admittance Controller

Admittance control is a widely used model in PHRI, it establishes the dynamic relationship between external force and the motion of the robot. Assume one-dimensional admittance equation in joint space is written as follows:

(5) $$\tau_{h}=m{\ddot{\theta }}_{d}+c{\dot{\theta }}_{d},$$

where *τ*_h_ is the torque exerted by human, *m* is the virtual mass, *c* is the virtual damping, and *θ*_d_ is the desired angular position. The trajectory to be followed by the manipulator can be prescribed in this linear second-order relationship form. The dynamic behavior of the manipulator and the effectiveness of the interaction are determined by the admittance parameters, namely the virtual mass and the virtual damping, and the dynamic model can be explained as moving an object with the virtual mass *m* in a virtual viscous (damping) *c* environment when a torque *τ*_h_ is applied to the object.

Compared with the virtual damping, the effect of virtual mass in the physical human-robot interaction can be neglected [12]. As a result, we regulate the dynamic behavior of the admittance model in the form of combining variable virtual damping with constant virtual mass. The virtual damping determines the response level of the manipulator to the external force, low virtual damping can reduce the force exerted by humans, however, it also reduces the positioning accuracy of the tasks. On the contrary, the operator can perform high accuracy movement with high virtual damping but it requires more human effort. In order to overcome the shortcomings of the fixed admittance model, a variable admittance control model with a suitable damping modulation strategy is typically required—e.g., at the beginning of the task, the virtual damping should be reduced for high velocity motion—and a high virtual damping is necessary for accurate positioning at the end of the movement. In this paper, the applied torque in the joint is used to infer the human intentions, which is a direct way to reflect the human intentions, and the turning of the damping can be defined as follows:

(6) $$c_{e}=c_{\min}+(c_{\max}-c_{\min})e^{-k_{e}|\tau_{h}|},$$

where *c_e_* is the output of the human intention estimator, *k*_e_ is a constant coefficient, *c*_min_ and *c*_max_ are the boundary values of the virtual damping, respectively. When the magnitude of the torque increases, it can be interpreted as a human intention to change the current motion state rapidly. In this situation, the virtual damping should be low for reducing the virtual inertia of the system. On the contrary, when the magnitude of the torque decreases, it means that the operator intends to decelerate or maintain the movement. In order to avoid over response, a high virtual damping is needed.

### 3.2. Damping Modulator Based on FSL

There are two critical characteristics for improving the feeling perceived by operators in physical human-robot interaction. One is the capability to respond the human intentions dynamically, which is solved by Human Intention Estimator as mentioned above. The other is the ability to enhance the comfort level during the task execution. Although the Human Intention Estimator can be used to deal with low positioning accuracy and hysteresis (high force required to change the motion state), the smoothness during task execution is not guaranteed. In order to improve the smoothness, it requires adaptation to individual behaviors during the manual guidance, and taking the human part of the controller into consideraton. Generally, we do not have the knowledge about the characteristics of individual behaviors, and it is very difficult to model. As a result, Fuzzy Sarsa(λ)-learning (FSL) is adopted to deal with the unpredictability of human behaviors by online learning in this paper. In [32], a minimum jerk trajectory model is used to learn the appropriate damping for effective cooperation. This model is proposed in the point-to-point linear motion of the human in Cartesian trajectories, and is not applicable for the joint space [21]. In our study, we take the square of jerk as a smoothness performance index to optimize the virtual damping estimated by the Human Intention Estimator during the interaction. The performance index is defined by the following objective function:

(7) $$J=\int_{0}^{T}{\dddot{\theta }}^{2}(t)dt,$$

where $\dddot{\theta }(t)$ is the jerk of the human hand, and *T* is the duration of motion. Smaller values of *J* indicate better physical human-robot interaction performance.

In general, reinforcement learning problems are discrete time-dynamic problems. The agent can only get a discrete state perception and trigger discrete actions in traditional model-free reinforcement learning. More specifically, the agent and the environment interact at each of a sequence of discrete time steps [30]. However, the problems have large or continuous state spaces, such as physical human-robot interaction, the traditional reinforcement learning is limited by the curse of dimensionality. To overcome the limitation, FSL is adopted in the real-world problems, in which fuzzy rules are used as a parameterized approximator to represent the continuous state space and integrate discrete actions as a continuous action output [33,34].

**Figure 2:**
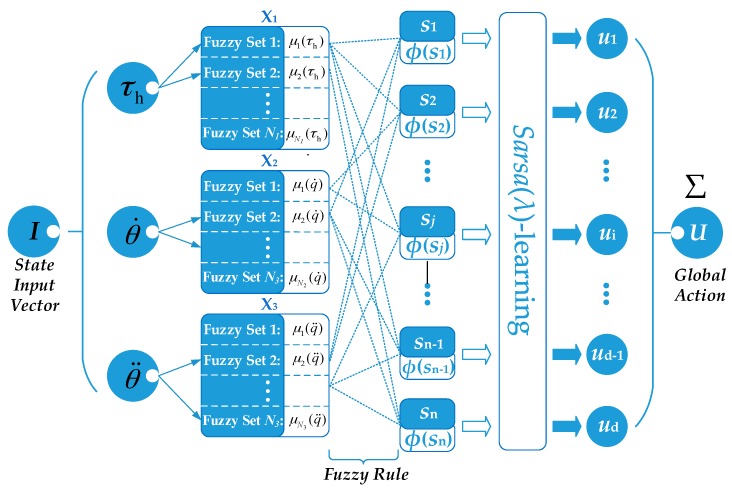
Flow chart of FSL.

In FSL, as shown in Figure 2, the continuous state spaces are divided by fuzzy sets which are described by the membership functions in the space of the input state variables (X_i_). The agent visits a fuzzy state set partially in the fuzzy state representation, and it depends on the normalized T-Norm:

(8) $$\varphi (s_{j})=\prod_{i=1}^{m_{v}}\mu_{j}(I_{i})/\sum_{k=1}^{n_{r}}\prod_{i=1}^{m_{v}}\mu_{k}(I_{i}),$$

where *s_j_* is the *j-*th fuzzy state component, *φ*(*s_j_*) is the *j-*th activated fuzzy rule value that indicates the proportion of the state components *s_j_* in the current state partition, *μ*(*I*) is the membership degree of the state variable *I*, *m*_v_ is the number of state variable and *n*_r_ is the number of the activated fuzzy rule. For the movement of the manipulator in joint space, the human intended torque *τ*_h_, the joint angular velocity $\dot{q}$, and the angular acceleration $\ddot{q}$, are selected as state variables. The action set of the agent is a discretization of action space which consists of the compensation value of the virtual damping, i.e., *u =* {*u_1_*, … ,*u_d_*}. At each time step *t*, the membership degrees of the state variables are calculated by triangular membership function, and more than one fuzzy rule can be active at the same time. Each activated fuzzy rule has a fuzzy state component, an optimal discrete action, and a weight associated with this state-action pair. The weight *w*(*s_j_*,*u_j_*) indicates the quality of a given action *u_j_* with respect to a state component *S_j_* , which is an estimation of how good it is for the agent to perform the action *u_j_* in the current state *S_j_* . It is used to select the optimal discrete action in each fuzzy state component. Update the eligibility trace according to Equation (9), and the weight is defined by Equation (10):

(9) $${\tilde{e}}_{t}(s,u)=\{\begin{array}{ll} \gamma \lambda {\tilde{e}}_{t-1}(s,u)+\varphi (s_{j}) & if\;s=s_{j}\;and\;u=u_{j}\\ \gamma \lambda {\tilde{e}}_{t-1}(s,u) & otherwise \end{array},$$

(10) $$w_{t+1}(s_{j},u_{j})=w_{t}(s_{j},u_{j})+{\tilde{\delta }}_{t}{\tilde{e}}_{t}(s_{j},u_{j}),\;{\tilde{\delta }}_{t}=r(S_{t},U_{t})+\gamma Q_{t}(S_{t+1},U_{t+1})-Q_{t}(S_{t},U_{t}),$$

where *Q_t_*(*s_t_*,*u_t_*) is the action value function which is computed as a weighted sum of the activated fuzzy rule value, and the general form is defined by Equation (11), *r*(*S_t_*,*U_t_*) is the reward received between two adjacent input states, it depends on the last action which has been performed by the agent in the previous state. Because the reinforcement learning is an algorithm to find a policy which seeks to maximize the accumulated reward in an episode, the general form of the reward is designed as Equation (12) (a negative sum of squares).

(11) $$Q_{t}(S_{t},U_{t})=\sum_{j=1}^{n}w_{t}(s_{j},u_{j})\varphi (s_{j}),$$

(12) $$r(S_{t},U_{t})=-\sum_{\Delta t}{\dddot{\theta }}^{2},$$

where *Δt* is the time interval between two learning steps (i.e., the sampling period of the algorithm). The weights determine the probability of choosing the local action *u* in each fuzzy rule. Update the Boltzmann exploration strategy (Equation (4)), and get the form as follows:

(13) $$P(u_{j}|s_{j})=\frac{e^{w_{t}(s_{j},u_{j})/\eta }}{\sum_{i=1}^{d}e^{w_{t}(s_{j},u_{i})/\eta }}$$

In Fuzzy Sarsa(λ)-learning, the input state space is divided by fuzzy rules. At each time step, the agent visits a fuzzy state which is represented by several activated fuzzy rules, and the activation degree of each activated fuzzy rule is indicated by the normalized T-Norm *φ* (Equation (8)). Then the local actions selected in each fuzzy state component are weighted by the corresponding activation degrees and constitute a continuous global action *U_t_* which is performed by the agent to compensate the virtual damping *c_e_*, and the output of the Damping Modulator *c_e_* is expressed as follows:

(14) $$c_{r}=U_{t}(S_{t})=\sum_{j=1}^{n}u_{j}\varphi (s_{j})$$

At last, the final virtual damping *c* provided to the admittance controller can be obtained by Equation (15).

(15) $$c=c_{e}+c_{r}$$

According to the above analysis, the function of fuzzy rules is to realize the perception of the continuous state at the input of reinforcement learning and integrate discrete local actions as a continuous global action output. During the online learning and training process, the agent learns how to perform actions which maximize the performance index (Equation (7), the accumulated reward in PHRI) in the long run.

## 4. Experimental Evaluation

In this part, the experimental evaluation of the proposed variable admittance model is conducted on our minimally invasive surgical manipulator. First, the characteristics of the minimally invasive surgical robot in physical human-robot interaction are illustrated according to the special structure of the manipulators, and then, in order to demonstrate the effectiveness of the proposed method, some comparative experiments between different admittance models are performed in the following, and a questionnaire is provided to the participants after the experiments.

### 4.1. Minimally Invasive Surgical Manipulator

**Figure 3:**
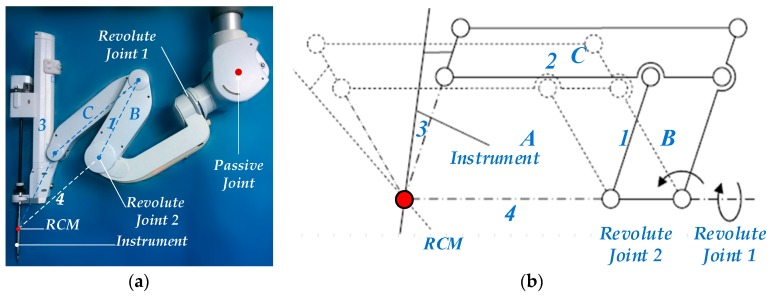
(**a**) Minimally invasive surgery manipulator; (**b**) The structure diagram of RCM.

As shown in Figure 3a, the minimally invasive surgical manipulator consists of three parts, i.e., the passive adjustment joint, the RCM (Remote Centre of Motion) kinematic mechanism, and the minimally invasive surgical instrument. The RCM structure can realize arbitrary rotation around a fixed spatial pivot point (RCM), as shown in Figure 3b, lever 1, 2, and 3 are driven by the revolute joint 2 and can rotate around the RCM point via the geometrical constraints among the parallelogram A, B, and C. Meanwhile, the plane mechanism is driven by a revolute joint (Revolute Joint1) whose axis passes through the RCM point. The passive adjustment joint is used to adjust the position of the RCM. During the operation, the passive joint must be locked, and the minimally invasive surgical instrument is driven by the RCM kinematic mechanism.

As discussed above, the position of the RCM is invariant during the configuration adjustment. In contrast to the industrial robot teaching and playback process, the concern of the medical robots in physical human-robot interaction is not the trajectory of the end-effector in Cartesian space but the attitude adjustment of the robot link on a joint level. The purpose of the adjustment is to enlarge the coverage rate of the workspace in the operation area and reduce the intraoperative interference among manipulators. Therefore, an active compliance control along the entire robot structure is required. For this reason, the torque sensors are installed in each revolute joint, and each robot link can be adjusted independently according to the measured torque in its attached joint. During the interaction, measuring the torques in the joints is advantageous for direct measuring and control. Moreover, the manipulator can be operated in an arbitrary position, and the attitude adjustment is not constrained by the place of application of torque.

### 4.2. Experimental Design

In the evaluation of the proposed variable admittance model, three sets of experiments are performed. In the first two series of experiments, a performance comparison between the proposed model and the fixed admittance model is conducted to verify the self-adjustment capacity during the task execution, and then the smoothness characteristics of this model are evaluated by comparison with a torque based variable admittance model (*c = c*_e_) in the third series of experiments. Finally, a questionnaire is given to each participant for rating the control approaches by intuitiveness.

**Figure 4:**
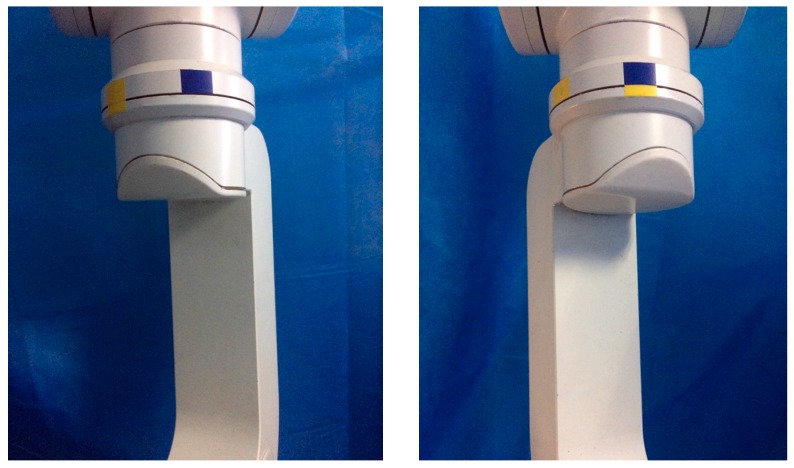
Target positons in the experiments.

The experiments are performed by eight participants aged from 22 to 39, and all the participants have no previous experience in physical human-robot interaction. Before the experiment, the participants are instructed about the task and have a few minutes to interact with the manipulator. In the experiments, the participants are asked to grab the robot link in any way they prefer, and guide a rotational movement of the joint between two targets in a single direction. As shown in Figure 4, the target positions are indicated to the participants by color ribbon. For each participant, three comparison experiments are conducted with four different control approaches in joint space, i.e., two kinds of fixed admittance control with different virtual damping, a torque based variable admittance control, and a variable admittance control based on FSL. In our study, we define the movement from the blue ribbon (π/6) to the yellow ribbon (−π/6) as Motion 1, and the opposite movement is regarded as Motion 2 which is treated as an episode of the task in reinforcement learning. FSL regulates the damping to maximize the accumulated reward (7) in an episode, and then the episode is repeated consecutively until reinforcement learning algorithm convergence.

The performance of the proposed variable admittance controller is measured on our experimental minimally invasive surgical manipulator based on TwinCAT real-time control system with a sampling period of 0.4 ms. The torque measured from the torque sensors are filtered by a second-order low-pass filter before it is introduced as the input of the admittance controller. During the physical interaction, the human perception is mainly influenced by the virtual damping, and the selection of the virtual mass is related to the stability of the system. For avoiding vibration, the virtual mass is set to a constant value (*m =* 0.25 kg) which is found experimentally. In FSL, because the reward received between two learning steps is always negative (a negative sum of squares in Equation (12)), the weight can be initialized to an upper bound to stimulate exploration. When the Boltzmann exploration strategy is adopted for the action selection, the weight values will gradually decrease with the process of learning and the actions selected will rarely have greater chance to be chosen. For each state-action pair, the eligibility trace $\tilde{e}(s,u)$ and the weight *w*(*s*,*u*) are initialized to zero at the beginning of the learning. In order to capture the continuous state input, a fuzzy partition is defined over the state space. Each state variable is uniformly partitioned by five fuzzy sets in state variable space, and then 125 fuzzy rules are generated by this state partition. The discrete action consists of five compensation values, i.e., *u* = {0, 0.003, −0.003, 0.006, −0.006}. The space of state variable X_i_ and the parameters of the proposed algorithm are listed in Table 1 and Table 2.

### 4.3. Experimental Results and Discussion

**Figure 5:**
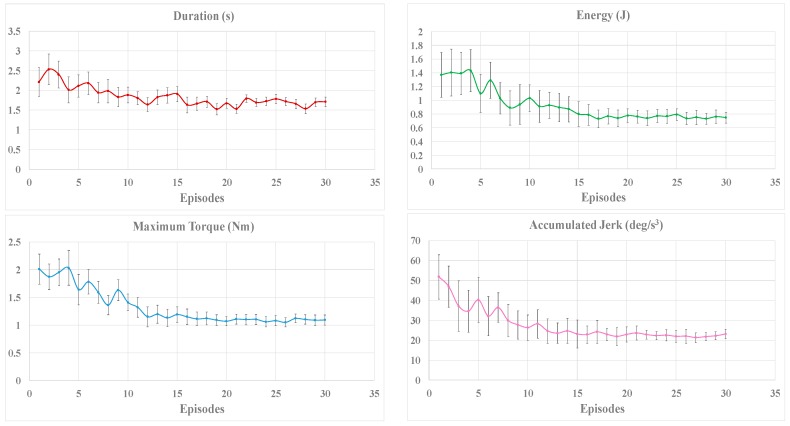
The trend of the evaluation criteria in the experiments.

In order to show the whole online learning process, the applied torque, the positioning accuracy, the transferred energy, and the duration of the movement are recorded individually for each participant during the training. The positioning accuracy is measured by the maximum distance after removing the interaction torque, and the energy transferred from the operator to the robot can be calculated by integrating the applied torque over the angle travelled. The mean value and standard deviation of the evaluation criteria in each episode for all the participants are illustrated in Figure 5.

As shown in Figure 5, the magnitude of the reward decreases gradually and stays in a stable value with small fluctuation after 15 episodes. Theoretically, the reinforcement learning algorithm can converge to an optimal solution through sufficient training, which may require much more time to achieve. However, as shown in Figure 5, the improvement of the evaluation criteria may be small or even imperceptible if the training continues. As a result, for the purpose of reducing time consumption, it is possible to stop the training process when the fluctuation of reward is stable and the reward is under an expected threshold.

#### 4.3.1. Contrastive Verification

**Figure 6:**
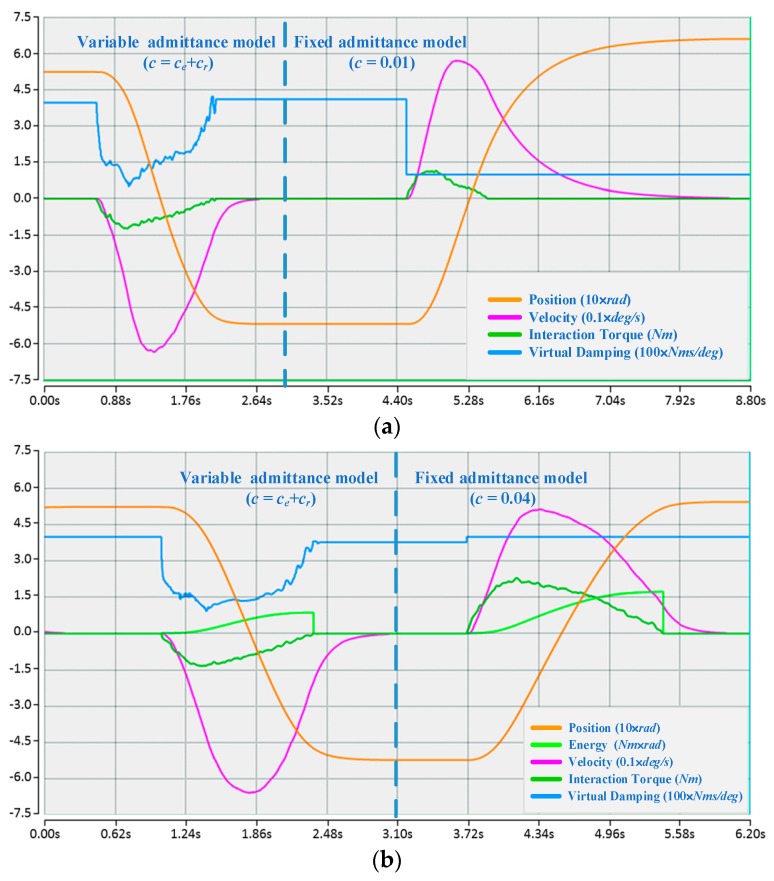
(**a**) Compared with low fixed admittance models; (**b**) Compared with high fixed admittance models.

**Figure 7:**
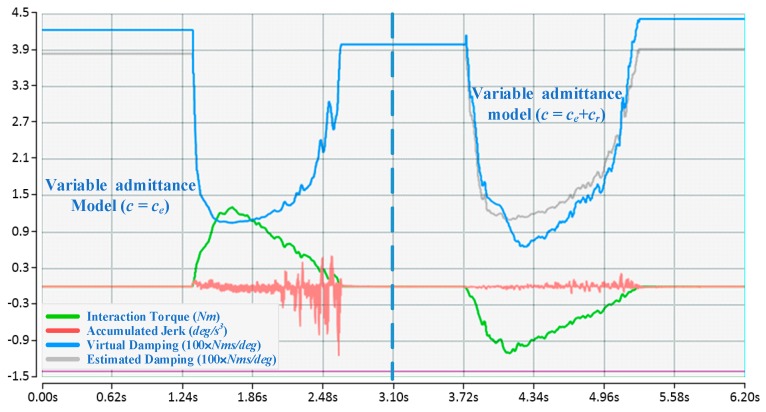
Compared with variable admittance model without damping adjustment ($c=c_{e}$).

**Figure 8:**
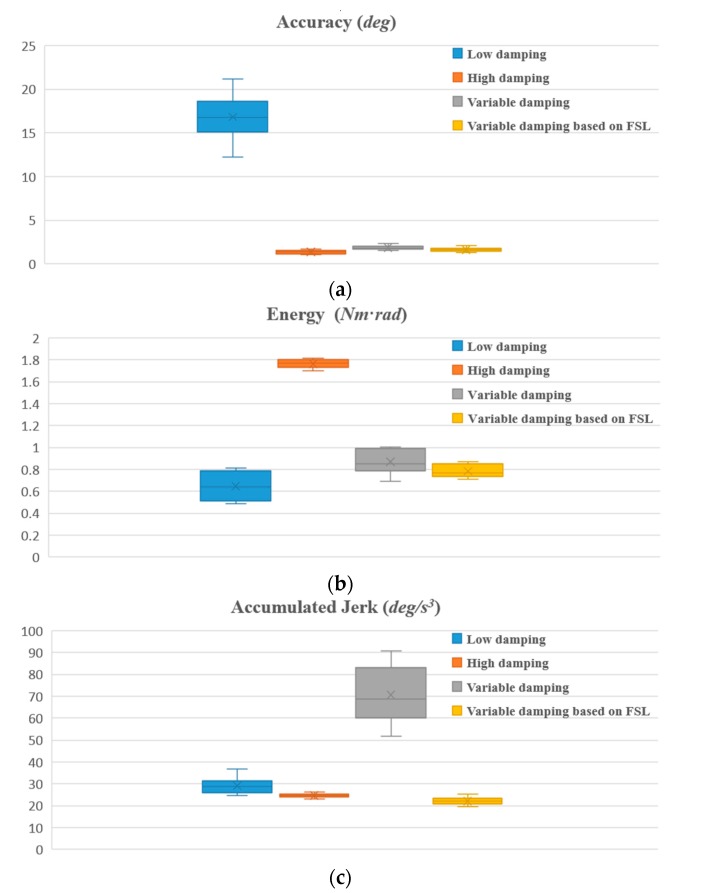
(**a**) Accuracy; (**b**) Energy transferred from the operator to the robot; (**c**) Accumulated jerk in the episode.

For each participant, when the proposed algorithm converges to an approximately optimal strategy, two comparison experiments with different fixed admittance models are first performed, the state of motion and the reward in an episode are depicted in Figure 6, and then a comparison with the variable admittance model based on torque is conducted as shown in Figure 7. The derived evaluation criteria of all participants in three sets of experiments are illustrated in Figure 8. Assisted by the proposed variable admittance model, the participants perform the experiments in better positioning accuracy than the fixed admittance model with low virtual damping (Figure 6a), the mean value of the maximum distance after removing the interaction torque is decreased by 90.3% (Figure 8a) and very close to that of the high virtual damping admittance model (Figure 7b), while the energy applied by the operator is reduced to 44.3% (Figure 8b) relative to the high virtual damping admittance model. In addition, analysis of the evaluation criteria compared to the variable admittance model based on torque indicates that the smoothness of the cooperation is improved significantly (Figure 7), and the mean value of the accumulated jerk in an episode is reduced to 31.4% (Figure 8c) relative to the variable admittance model based on torque. The low virtual damping admittance controller makes the manipulator much more sensitive to the interaction torque, which is one of the reasons for large fluctuation in the evaluation criteria. The uncertainty of the human operation and the dynamics change of the manipulator enlarge the jerk of the produced movements in variable admittance model.

#### 4.3.2. Questionnaire for Comments

In the physical human-robot interaction, since the human is part of the physical human-robot interaction controller and plays a crucial role in the overall performance, besides the experimental data, the subjective comments provided by operators are also used for evaluation. For this purpose, a questionnaire is given to the participants after the specified task execution, which is used to rank the feeling perceived from 1 (poor) to 3 (excellent) in terms of intuitiveness. In our study, the sense of being in control and the naturalness of the motion are used to describe the characteristics of the behaviors in PHRI. The naturalness indicates the degree of similarity of motion control in daily life, and the sense of being in control can be interpreted as a rapid response to change the motion state easily according to human intention.

**Figure 9:**
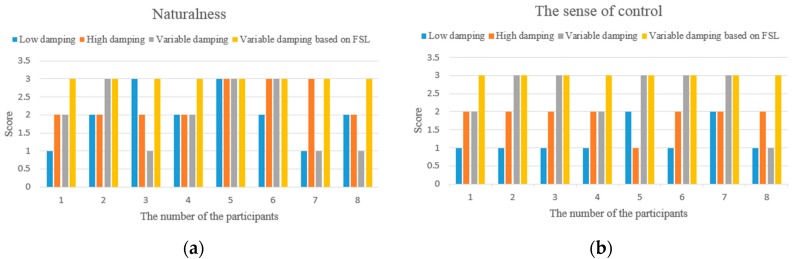
(**a**) The score of naturalness; (**b**) The score of sense of control.

The task executed is designed to correspond to a typical pose adjustment action for the manipulator. e.g., from the yellow ribbon to the blue ribbon (in Figure 4) as described in Section 4.2, and the passed angle is approximately π/3. For each participant, when the training process is over (the proposed algorithm converges to an approximately optimal strategy), four individual tests are asked to be performed repeatedly with four different controllers (fixed low damping, fixed high damping, variable damping based on force, and variable damping based on FSL) in a random order. The participants do not know which controller is used in the current operation, and are told about the definition of the studied behavior characteristics in PHRI before the experiments. Finally, the scores of the characteristics for each participant are illustrated in Figure 9.

## 5. Discussion

In this paper, an online learning strategy is proposed to get natural and intuitive physical human-robot interaction. Actually, the learning procedure of the interaction is bidirectional. When the robot is training to adapt to the individual behaviors, the operator also can receive the information from the robot that may influence the operator unconsciously. For the purpose of minimizing the influence in the training procedure, each participant has enough time to interact with the robot. The experimental results obtained in this paper are under the assumption that the participants have sufficient knowledge for the learning process and repeat the same action as much as possible in each training step.

As shown in Figure 5, the deviations of the evaluation criteria tend to stabilize with the increase of training time. At the first 10 episodes, the magnitude of the jerk varies greatly for different participants, which caused by individual behaviors. The Fuzzy Sarsa(λ)-learning tries to find an appropriate strategy for maximizing the rewards. During the next 10 episodes, the strategy is improved constantly until convergence, the energy required, the time consumed, and the accumulated jerk in the training process are significantly decreased relative to the initial values. It is proven that the online learning strategy can significantly improve comfort level during the task execution for different people. Figure 5 also shows the capability of rapid convergence of the proposed algorithm (within 20 episodes), which is important for the adaptation of different operators.

As shown in Figure 6 and Figure 7, the proposed variable admittance strategy is able to estimate the human intentions according to the state input. At the beginning of the interaction process, it provides low virtual damping to the admittance controller for eliminating the feeling of motion delay, and increases the damping value to improve the positioning accuracy at the end of the interaction. During the task, it also helps the operators to maintain the smoothness of the movement. From the results of the comparative experiments (as shown in Figure 8), it can be seen that the variable admittance controller based on FSL has good positioning accuracy and low energy consumption (close to the low virtual damping controller) during the task. The variable admittance controller proposed in this paper can not only overcome the limitation of the fixed admittance model but also improve the smoothness of the traditional variable admittance model.

From the aspect of intuitive perception, participants comment that it is easy to accelerate but difficult to perform fine positioning (caused by over response) with the low variable admittance controller. On the contrary, it is easy to perform fine positioning in the high admittance case, however, high human effort is required to change the motion state. Although the traditional variable admittance controller can find a compromise in these two cases, but the participants feel the interaction is not smooth enough. According to the questionnaires and the comments provided by the participants (as shown in Figure 9), all of them acknowledge that the variable admittance controller based on FSL has the best interactive experience. After several repetitions of the online training step, each participant can perceive the assistance provided by the agent during the task. Some participants pointed out that it is just like moving an object in liquid when they operate the manipulator with this model.

## 6. Conclusions

The main objective of this paper is to find an appropriate approach to regulate the virtual damping of the variable admittance model for an intuitive physical human-robot interaction in joint space. A variable admittance algorithm based on Fuzzy Sarsa(λ)-learning is proposed to compensate the error of the virtual damping caused by improper model parameters or the changing dynamics of the robot. The algorithm has the property of self-adaptation and does not require any environment model. The experimental data and questionnaire indicate that the proposed model is able to regulate the damping of the admittance controller appropriately for improving the positioning accuracy and reduce the energy required, and is much more intuitive in comparison with the traditional admittance controller.

The introduction of reinforcement learning will be the main trend of the research for physical human-robot interaction in the future. The basic framework of the variable admittance model based on reinforcement learning for the minimally invasive surgery robot presented in this paper, due to the independent of the model in joint space, can be easily extended to the entire joint configuration of the manipulator for other areas of application. The number of the discrete action is given by experience at present. The effect of the number for smoothness is not apparent but too many discrete actions will greatly increase the time consumption of the algorithm convergence. In order to improve the variable admittance strategy and eliminate the unreasonable fuzzy rules settings, more research work is required for fuzzy parameter optimization, such as the interval of discrete actions and the shape of the MFs, which is our future research direction for the proposed framework.

## Figures and Tables

**Table 1:** The parameters of reinforcement learning.

*γ*	*α*	*λ*	*η*	*Δt* (s)
0.9	0.03	0.95	85	0.004

**Table 2:** The universe of discourse of the state variable and the parameters of the intention estimator.

X_1_ (Nm)	X_2_ (deg/s)	X_3_ (deg/s^2^)	*k*_e_	*c*_min_ (Nms/deg)	*c*_max_ (Nms/deg)
−2.5 ~ 0.0	−8.5 ~ 0.0	−4.5 ~ 4.5	3.06	0.01	0.04
